# Fantastic stewards and where to find them: a descriptive study of the heterogeneous disparity in physician–pharmacist–nurse distribution among the infectious disease workforce

**DOI:** 10.1017/ash.2025.10287

**Published:** 2026-01-26

**Authors:** Yuuka Uesugi, Satoshi Kitaura, Koh Okamoto

**Affiliations:** 1 Department of Clinical Training, Aso Iizuka Hospital, Fukuoka, Japan; 2 Department of Infection Control and Prevention, The University of Tokyo Hospital, Tokyo, Japan; 3 Department of Infectious Diseases, The University of Tokyo Hospital, Tokyo, Japan; 4 Department of Infectious Diseases, Institute of Science Tokyo Hospital, Tokyo, Japan

## Abstract

**Background:**

Infectious disease (ID) care involves a diverse range of professionals, yet the shortage and geographical disparity of ID physicians, among other professionals, remain underexplored. This cross-sectional descriptive study aimed to elucidate the distribution of ID physicians, infection control (IC) pharmacists, and IC nurses in Japan, focusing on the interrelations among the ID workforce within medically relevant geographical units.

**Methods:**

Publicly available data from 335 secondary medical areas (SMAs) in Japan, with a population of 125 million, were analyzed. Workforce distribution was assessed using the Gini index to quantify inequalities and spatial clustering across the SMAs per capita, per hospital bed, and per unit area. χ2 test was used to assess the association of hospital characteristics with the presence of each professional.

**Findings:**

The research subjects were 1,729 ID physicians, 1,371 IC pharmacists, and 2,657 IC nurses, whose workplace data were available in Japan as of 2023. The Gini coefficients for the densities of ID physicians, IC pharmacists, and IC nurses per 100,000 people were 0.46, 0.34, and 0.28, respectively. The density of the ID workforce per unit area (1,000 km^2^) showed a positive correlation ( > 0.8) with any combination of ID physicians, IC pharmacists, and IC nurses. A total of 186 SMAs (56%) had at least one member from each professional group and 13 (3.9%) lacked staff from all three.

**Conclusions:**

The substantial variation in ID workforce composition across SMAs suggests opportunities for both regional and national policy and identifies new avenues for improving access to ID care.

## Introduction

Antimicrobial resistance, emerging and reemerging infectious diseases (IDs), and a rising number of immunocompromised patients stress the need for accessible ID care. Most recently, the coronavirus disease 2019 (COVID-19) pandemic has resurfaced vulnerability in ID specialist care owing to the shortage and regionally biased distribution of ID physicians.^1^ Diverse ID specialist care, from consultation to infection control (IC) and antimicrobial stewardship, is now provided by multidisciplinary members including ID physicians, pharmacists, nurses, and other professionals.^2^

Access to care remains a significant challenge, and medical professionals available in close proximity would be desirable. The current supply of ID physicians suggests both shortage and geographical disparity.^3^ This highlights an important opportunity: the integration of co-medical professionals, such as IC pharmacists and nurses, can effectively synergize with ID physician services. Evidence from infection prevention programs shows that IC nurses prevent urinary infection among patients who received surgery.^4^ The availability of IC pharmacists and nurses, and the collective capacity for multidisciplinary care with ID physicians, however, are significant knowledge gaps.

Japan faces a unique set of challenges due to its demographic and healthcare system characteristics, including but not limited to low physician density (2.6 per 1,000 population), high nurse density (12.2 per 1,000), and a high 12.5 hospital beds per 1,000 population.^5^ The country is undergoing rapid population aging and is expected to face a growing burden of IDs among the elderly.^6^ While certification of ID-related professionals involves stringent requirements, the growing demand for ID care is a significant concern in maintaining access to ID care. Such imbalance contributes to gaps in multidisciplinary ID management, underscoring the need for a new dimension to understanding ID care delivery. Taken together, Japan offers a particularly informative setting in which to examine the detailed distribution of the ID workforce, given the high demands for ID care.

In this study, we aimed to characterize the uneven distribution of ID physicians and other ID professionals, including IC pharmacists and IC nurses, in Japan by utilizing three specific evaluation metrics: per capita, per hospital beds, and per area. Furthermore, we investigated the allocation of the ID workforce according to hospital characteristics. Through these proof-of-concept analyses, we visualized the presumed “gaps” through the perspective of collaborative ID care, thereby offering a framework to visualize workforce imbalances in societies facing high burden of IDs.

## Method

### Study design and setting

This cross-sectional study was conducted to describe the distribution of physicians, pharmacists, and nurses working as ID specialists in Japan, using publicly available data on archived lists of names and workplaces of ID specialists. All data were retrieved from each professional organization’s website in May 2023. In Japan, with a population of 125 million in 2022,^7^ there are approximately 343,000 registered physicians,^8^ 324,000 registered pharmacists^8^ and 1.7 million registered nurses^9^ nationwide. Board certification as an ID physician by the Japanese Association of Infectious Diseases requires a minimum of six years of clinical training in ID, including residency and three presentations at academic conferences, in addition to passing the examination.^10^ Certification as an IC pharmacist by the Japanese Society of Hospital Pharmacists requires membership in a relevant organization, publication of at least one academic manuscript on IC, passing the certification examination, and obtaining a recommendation from the hospital director or facility head.^11^ IC nurse certification by the Japanese Nursing Association requires a minimum of five years of work experience and completion of a specialized course in ID lasting six months to one year.^12^ No individual holds either qualification simultaneously. All certifications must be renewed every five years.

### Outcomes

Three analyses were performed to provide a multidimensional perspective on the distribution of physicians, pharmacists, and nurses who work as ID specialists. First, we calculated the Gini coefficient for each professional group per capita, per hospital bed, and per unit area. Second, we investigated the correlation between the presence of each professional group and described the variation in the composition of each professional group according to Secondary medical area (SMA). Third, we evaluated the number of professionals in hospitals stratified by hospital size, the presence of an established antimicrobial stewardship program (ASP), university affiliation, and the availability of residency programs for physicians.

### Data sources for denominators

SMA was selected as the geographical analysis unit. Within each SMA, the total capacity of hospital (beds) providing general inpatient care is governed by the Medical Care Act, which considers population size and composition (including age, illness rates, cross-boundary patient flows, geographical factors, state of meeting daily life demands, and transportation conditions).^13^ There are 335 SMAs in Japan, which are geographic entities employed in healthcare planning and typically encompass multiple municipalities. The median population of each SMA is 225,000, with interquartile ranges (IQR) from 103,000 to 479,500.^14^

To calculate the number of professionals per capita, hospital, and area, we used the population, hospital beds, and area data for each SMA. Population and area data were obtained from the national land numerical information provided by the Ministry of Land, Infrastructure, and Transport.^14^ Publicly available data on the number of hospital beds per SMA were obtained from the Medical Facility Survey.^15^

Data on the number of beds per hospital was obtained from the regional healthcare information system published by the Japan Medical Association.^16^ In Japan, there are a total of 8,068 hospitals, of which 1,029 offer residency training programs.^17,18^ We used the open data list provided by the Japan Residency Matching Program, which provides information on hospitals providing residency programs, to analyze the current state of ID education for resident physicians In addition, publicly available data from the Healthcare Market Analysis Platform^19^ revealed hospitals that received Infection Control Improvement Fee and whether they had a full-time ID physician. Infection Control Improvement Fee in Japan is a medical fee system that allows hospitals with an established ASP to add a surcharge when patients are hospitalized.^20^ For the Infection Control Improvement Fee One, hospitals must establish Antimicrobial Stewardship Team constituted by full-time physician with at least 3 years of experience in IC (not limited to ID physicians), dedicated nurse with at least 5 years of experience in IC and completion of appropriate infection management training (IC nurse), dedicated pharmacist involved in infection prevention with at least 3 years of hospital work experience, dedicated clinical laboratory technologist with at least 3 years of hospital work experience involved in infection prevention.^20^

### Ethical consideration

This study used publicly available data, and all identifiable information was removed before data acquisition. The data used in this study did not require an institutional review board because it was considered outside the scope of the Japanese national guidelines for clinical research, the Ethical Guidelines for Medical and Biological Research Involving Human Subjects.

### Statistical analysis

The Gini index was used to represent the degree of variation in the distribution. The Gini index was originally used to measure income inequality within a specific group.^21^ The index ranges from zero to one, with a higher value indicating a more unequal distribution. The χ^2^ test was used for calculating the odds ratio (ORs) and confidence interval for ID professional availability by hospital characteristics. All analyses including χ^2^ test were performed using Python 3.10.2.,^22^ NumPy 1.25.2.,^23^ Pandas 2.0.3.,^24^ GeoPandas 1.0.1.,^25^ Shapely 2.0.6.,^26^ Matplotlib 3.8.0.,^27^ and Folium 0.19.2.^28^ through Google Colaboratory.^29^

## Results

### Number of infectious disease specialists

Overall, Japan had 1,731 ID physicians, 1,397 IC pharmacists, and 3,338 IC nurses as of May 2023. Workplace data were available for 99.9% (*n* = 1,729/1,731), 98.1% (*n* = 1,371/1,397), and 79.6% (*n* = 2,657/3,338). Considering Japanese population, we have 1.40 ID physicians, 1.12 IC pharmacists, and 2.67 IC nurses per 100,000 people, respectively.

### The Gini coefficient for each professional

**Figure 1.**
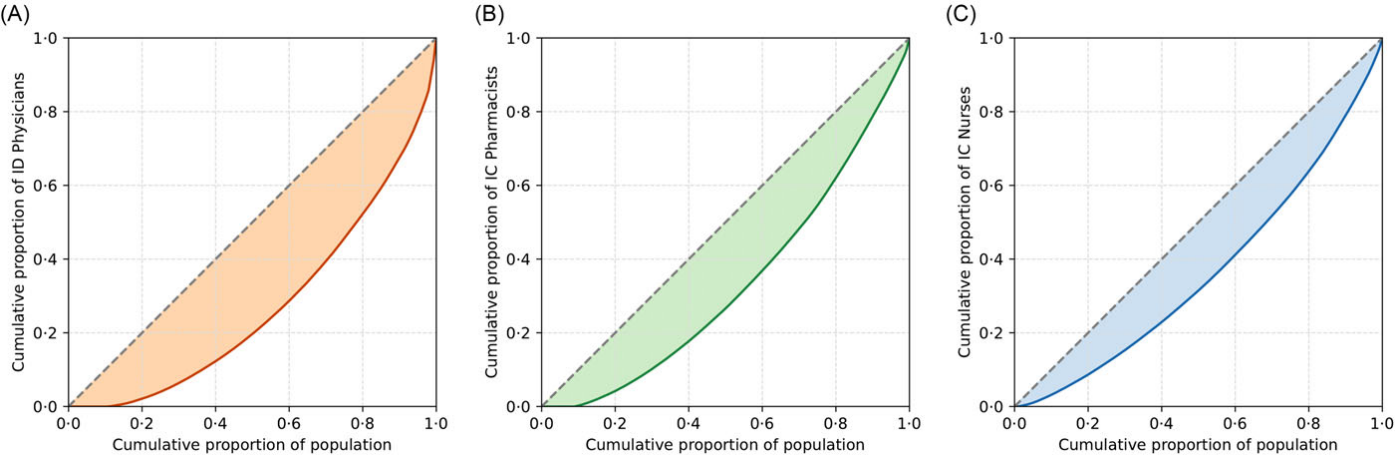
Lorenz curves and Gini coefficient. A: Japanese ID physician distribution per 100,000 people (Gini coefficient 0·46); B: Japanese IC pharmacist distribution per 100,000 people (Gini coefficient 0·34); C: Japanese IC nurse distribution per 100,000 people (Gini coefficient 0·28).

**Figure 2.**
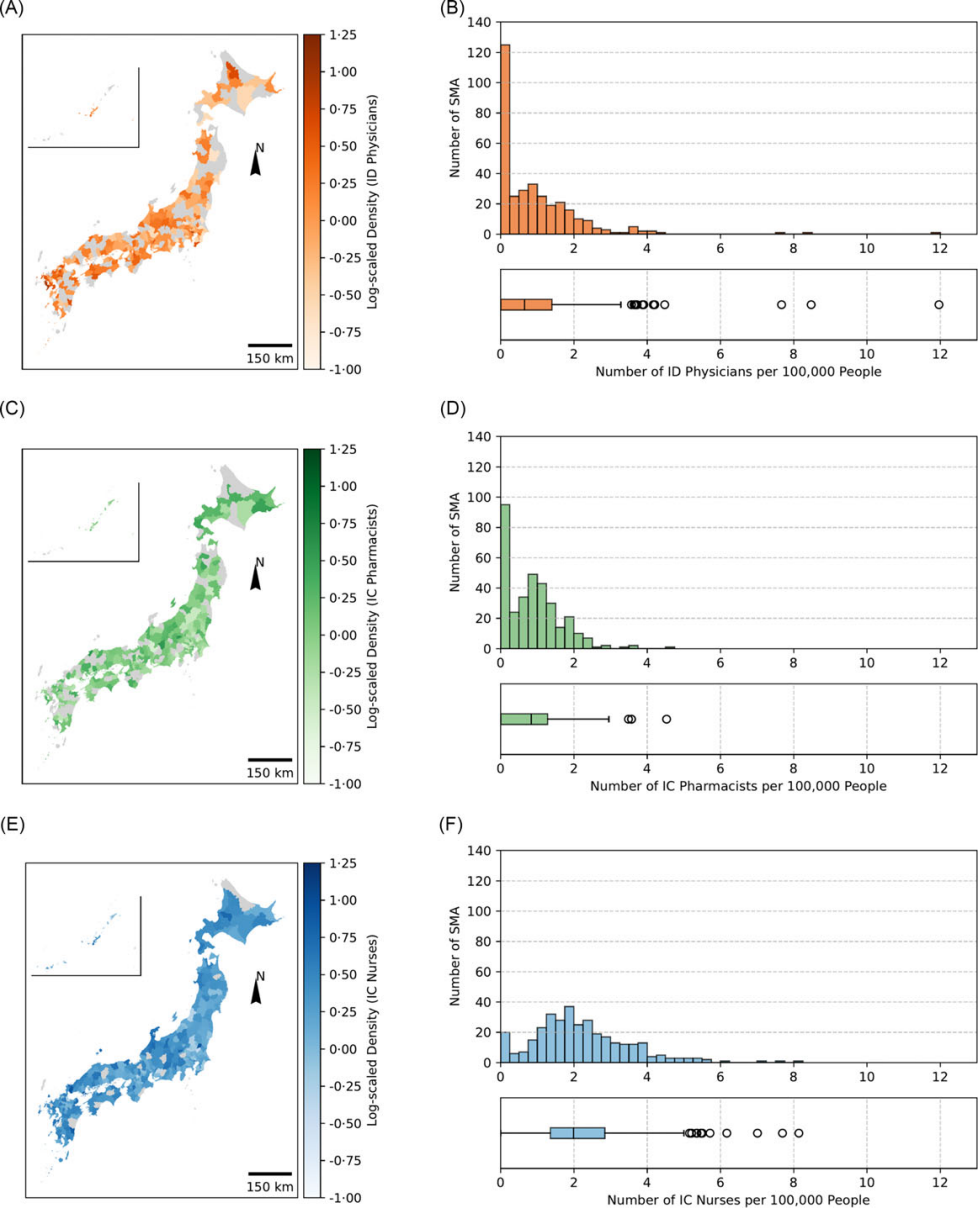
Geospatial mapping and distribution of Japanese ID workforce per 100,000 people. A: Japanese ID physician geospatial distribution, and B: Histogram and box-and-whisker plot describing density of ID physician; C: Japanese IC pharmacist geospatial distribution, D: Histogram and box-and-whisker plot describing density of IC pharmacist; E: Japanese IC nurse geospatial distribution, and F: Histogram and box-and-whisker plot describing density of IC nurse. The density of each professional is represented on figures B, D, and F as a color gradient based on values expressed using the common logarithm.

The Gini coefficients for the densities of ID physicians, IC pharmacists, and IC nurses per 100,000 people were 0.46, 0.34, and 0.27, respectively (Figure 1). Nearly 20% of the entire population and 35.2% of the SMAs (*n* = 118) had no ID physicians (Figure 2). The Gini coefficients for the densities of ID physicians, IC pharmacists, and IC nurses per 1,000 hospital beds were 0.52, 0.42, and 0.33, respectively (Supplemental Figures 1 and 2). The Gini coefficients per 100 km^2^ were 0.86, 0.79, and 0.70, for ID physicians, IC pharmacists, and IC nurses, respectively (Supplemental Figures 3 and 4).

### Correlation among the different ID workforce and compositional variation across SMAs

**Figure 3.**
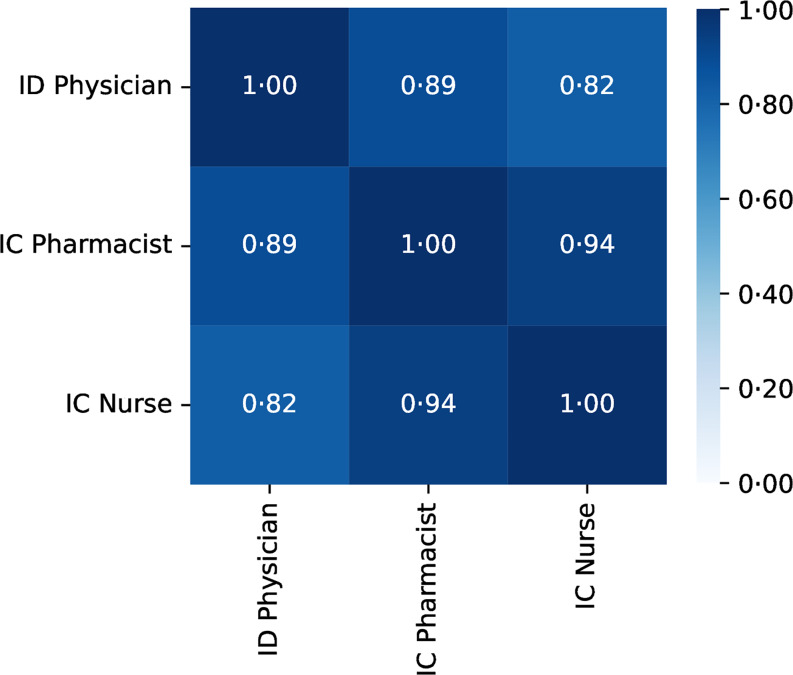
Correlation of regional distribution of Japanese ID workforce per area (1,00 km ^ 2).

**Figure 4.**
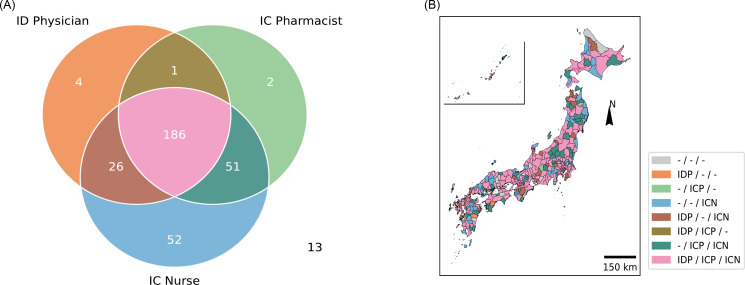
Overlap among different professionals among SMAs. A: A Venn diagram depicting overlap among SMAs with at least one ID physician/IC pharmacist/IC nurse. Thirteen SMAs did not have any ID physician/IC pharmacist/IC nurse and therefore were not included in the Venn diagram; B: Map color-coded by the presence of at least one ID physician, IC pharmacist, or IC nurse in each region.

The density of the ID workforce per unit area (100 km^2^) showed a positive correlation (> 0.8) for any combination of ID physicians, IC pharmacists, and IC nurses (Figure 3). The strongest correlation was observed between IC pharmacists and IC nurses (correlation coefficient: 0.94), whereas the weakest correlation was found between ID physicians and IC nurses (correlation coefficient: 0.82). We further investigated the heterogeneity in the distribution of the ID workforce by evaluating SMAs for the presence of at least one ID physician, IC pharmacist, or IC nurse (Figure 4). Approximately half of the SMAs (*n* = 186, 56%) had at least one member from each professional group, whereas a quarter (*n* = 78, 23%) lacked one of the three professional groups. Another 58 SMAs (17%) had only one professional group available within the area, and 13 SMAs (3.9%) had none.

### Number of ID professionals according to various hospital characteristics

**Figure 5.**
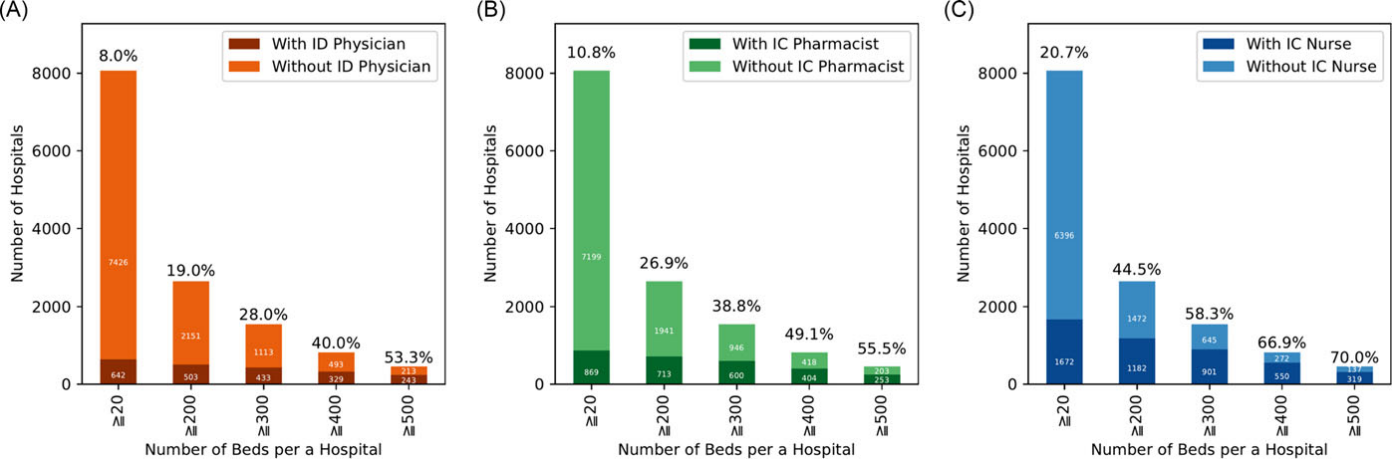
The number of hospitals with at least one member of the ID workforce, classified by hospital bed capacity. We included hospitals with ≥20 beds and excluded clinics with <20 beds. A: Number of hospitals with at least one ID physician. We included 642 hospitals with at least one ID physician and 7,426 hospitals without an ID physician, limited to hospitals with 20 or more beds. B: Number of hospitals with at least one IC pharmacist. We included 869 hospitals with at least one ID physician and 7,199 hospitals without an ID physician, limited to hospitals with 20 or more beds; C: Number of hospitals with at least one IC nurse. We included 1,672 hospitals with at least one ID physician and 6,396 hospitals without an ID physician, limited to hospitals with 20 or more beds.

We estimated odds ratios for the presence of at least one ID physician utilizing hospital bed size thresholds of ≥200, ≥300, ≥400, and ≥500 beds, with hospitals below each respective threshold serving as the reference group. Larger hospital size was associated with progressively higher odds of ID physician availability, with ORs (95% confidence intervals) of 8.87 (7.31–10.78), 11.8 (9.85–14.03), 14.78 (12.35–17.69), and 20.62 (16.72–25.44), respectively (Figure 5A). ORs for the presence of an IC pharmacist were calculated similarly, with ORs (95% confidence intervals) of 12.38 (10.33–14.84), 14.74 (12.57–17.29), 14.09 (11.94–16.64), and 14.15 (11.56–17.33), respectively (Figure 5B). ORs (95% confidence intervals) for the presence of an IC nurse were 8.07 (7.15–9.10), 10.42 (9.19–11.82), 11.04 (9.42–12.93), and 10.77 (8.74–13.27), respectively (Figure 5C). Of note, 1,343 (16.7%) hospitals received Infection Control Improvement Fee 1, and one-third of those (*n* = 448, 33.4%) had full-time ID physicians. Furthermore, when focusing specifically on teaching hospitals, 94.7% (125/132) of university hospitals had at least one ID physician, whereas only 39.2% (403/1,029) of residency programs had full-time ID physicians.

## Discussion

Our descriptive study showed a multidisciplinary distribution in relation to regional healthcare needs, which revealed a consistently skewed distribution of the ID workforce compounding shortage. Currently, there is no defined standard for optimal ID physician staffing which likely differ depending on country, the role of ID physicians in each health system and other factors including local epidemiology of IDs. Although limited data are available, the number of ID physicians per 100,000 people in the United States, Brazil, and South Korea have been reported as 1.76,^1^ 1.50,^7,30^ and 0.532,^7,31^ respectively. This study revealed an average 1.40 ID physicians per 100,000 people which is not in the lower range of the global ID physician supply. However, ID physicians were less distributed than general internists, whose Gini coefficient per 100,000 people in Japan was 0.203.^13^ The high Gini coefficient per hospital bed index also showed a consistently unequal distribution in Japan, which is characterized by a high number of hospital beds compared with other countries.^32^ The per-area index, a surrogate for geographical accessibility, visually confirmed dense clustering in heavily populated cities, such as Sapporo, Tokyo, Osaka, and Fukuoka. Moreover, only 28% of the hospitals with over 300 beds had full-time ID specialists, the criteria proposed by the Japanese Association of Infectious Diseases.^33^ In addition, ID physicians and IC nurses were not concurrently available in two-third of hospitals that received the infection Control Improvement Fee One.^20^ The situation in Japan, therefore, is characterized by a high regional disparity rather than a pure shortage.

The number of ID fellowship applications have been declining, further worsening the shortage of ID physicians in the United States.^34^ Early and effective exposure to the ID speciality is a global focus of research. Of the 1,029 hospitals in Japan that offer residency programs (out of 8,122 nationwide), more than 60% were found to have no ID physicians.^18^ Our results were in concordance with a previous study in Japan suggesting that half of the resident physicians were trained in hospitals without an ID consultation service, and a third with no opportunities to learn antimicrobial stewardship.^35^ Another large-scale questionnaire-based survey of Japanese resident physicians revealed that those working in hospitals with bedside ID consultations were more likely to know the concepts of ASP and be confident in antimicrobial prescriptions.^36^ Limited opportunities to experience specialist ID care is an important factor that could be addressed to attract medical students and residents to the ID workforce.

While the ID workforce was concentrated in several metropolitan areas, IC pharmacists and nurses were more dispersed than ID physicians based on population, hospital beds, and area. These results suggest that IC nurses and pharmacists are more readily present as frontliners for addressing ID specialist care in underserved areas, especially in the domain of IC and antimicrobial stewardship. In particular, IC nurses stood out as having a distinct distribution compared to other workforces. Even so, the absolute number of IC nurses remains low at only 0.45 per 100 hospital beds which is in contrast to the 0.8 IC nurses per 100 beds (IQR 0.38–2.06) in Europe.^37^ This may be explained by the relatively high number of beds per population.^32^ The oversupply of medical institutions and hospital beds is a potential hurdle in the standardization of care and is currently being addressed by both horizontal and vertical integration among healthcare facilities.^38^ These efforts are also relevant to IC pharmacists, in which the current gaps are likely filled by pharmacists without formal ID training as in other countries.^39^ Furthermore, simultaneous assessment revealed that only half of all SMAs had all three professionals (ID physicians, IC pharmacists, and IC nurses), with many SMAs having an imbalanced composition. These heterogeneities are major hurdles in multidisciplinary efforts, which are the cornerstones of a spectrum of ID specialist care, from diagnosis and treatment, antimicrobial stewardship, to IC and prevention.^2^ Given the discrete patterns of ID workforce shortage, a tailored approach is likely needed for each SMA. The advent of telemedicine and consultation is a potentially generalizable solution for bridging patients to ID specialist care.^40^

This study had several limitations. The degree of involvement in ID care remains uncertain among the professionals in each SMA. According to a national survey by the Japanese Ministry of Health, Labor, and Welfare, only 35.6% of ID physicians reported being primarily engaged in ID-related work.^8^ Additionally, we did not extrapolate the simultaneous distribution of professionals according to each institution. As Japan is characterized by the highest number of hospitals worldwide, ID workforce members listed within the same SMA may be employed across different facilities. Consequently, their co-presence within an SMA does not guarantee collaborative contributions to IC or antimicrobial stewardship within any single hospital or region. Even for institutions receiving Infection Control Fee 1, data on the co-presence of all three professionals were not readily available. Furthermore, the lower disclosure rate for IC nurses, relative to ID physicians and IC pharmacists, may introduce uncertainty in their distribution, highlighting an opportunity for improved data reporting in future studies. Second, this study focused on only three sectors of the ID workforce: physicians, pharmacists, and nurses. In reality, ID care is supported by various professionals, including but not limited to clinical microbiologists and hospital epidemiologists. Extending our proof-of-concept evaluation to the various key sectors of the ID workforce will enable a more accurate depiction of the supply of ID care and subsequent solutions.

## Conclusions

Simultaneous analysis of the ID workforce revealed a heterogeneous composition of key stakeholders among the clinically relevant geographical units. Exploring the relationship between the heterogeneous supply of the ID workforce and clinically relevant outcomes could help guide strategies for workforce distribution and collaboration, thereby enhancing IC measures according to regional healthcare needs. While workforce shortage is often attributed as a significant challenge for optimizing access to ID care, ongoing efforts are essential in bridging the different stakeholders of the ID workforce.

## Supporting information

10.1017/ash.2025.10287.sm001 Uesugi et al. supplementary material 1 Uesugi et al. supplementary material

10.1017/ash.2025.10287.sm002 Uesugi et al. supplementary material 2 Uesugi et al. supplementary material

10.1017/ash.2025.10287.sm003 Uesugi et al. supplementary material 3 Uesugi et al. supplementary material

10.1017/ash.2025.10287.sm004 Uesugi et al. supplementary material 4 Uesugi et al. supplementary material

## Supplementary material

The supplementary material for this article can be found at https://doi.org/10.1017/ash.2025.10287

## Use of artificial intelligence (AI) tools declaration

During the preparation of this manuscript, we used generative artificial intelligence tools to support the writing and editing process. Specifically, ChatGPT (OpenAI, version available as of 2025) and Paperpal (Cactus Communications) were used to assist with language refinement, sentence restructuring, and improvement of clarity and readability of the text. These tools were not used to generate original scientific content, interpret data, or draw conclusions. All scientific interpretations, analyses, and final wording were reviewed, verified, and approved by the authors, who take full responsibility for the content of the manuscript.
