# Supplementary figures and images for: Fantastic stewards and where to find them: a descriptive study of the heterogeneous disparity in physician–pharmacist–nurse distribution among the infectious disease workforce
Source: Antimicrob Steward Healthc Epidemiol. 2026 Jan 26;6(1):e29. doi: 10.1017/ash.2025.10287 (PMC12854878; doi:10.1017/ash.2025.10287)

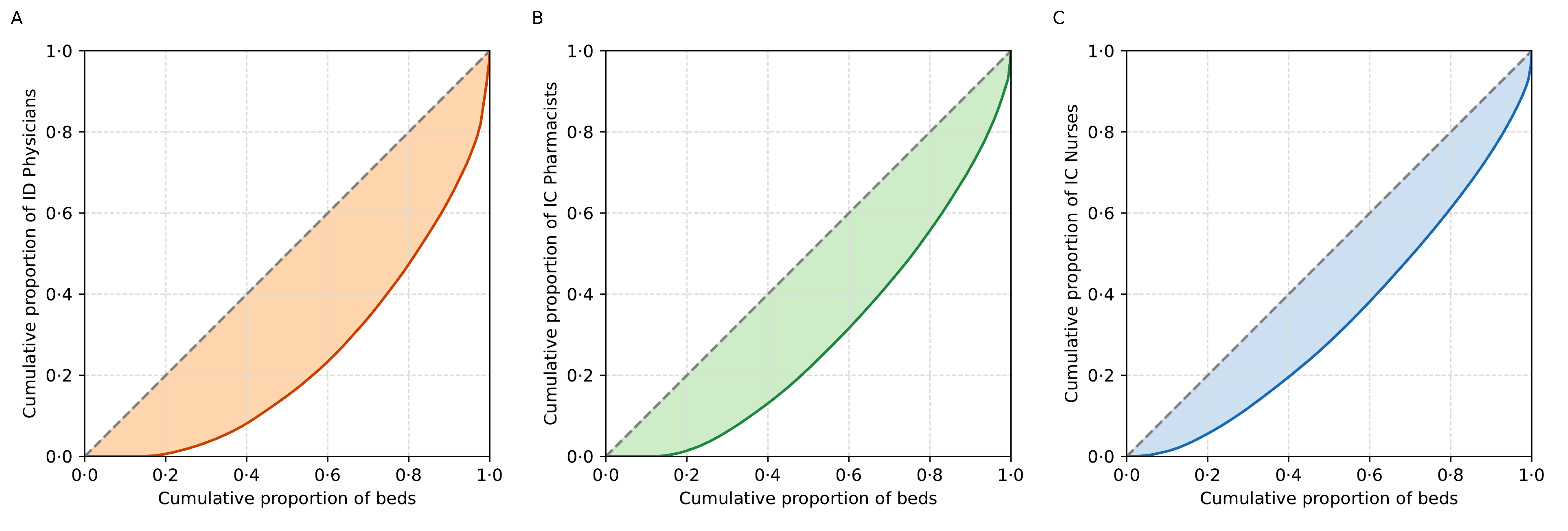

Supplement: Uesugi et al. supplementary material 1 — Uesugi et al. supplementary material [file S2732494X25102878sup001.tiff]

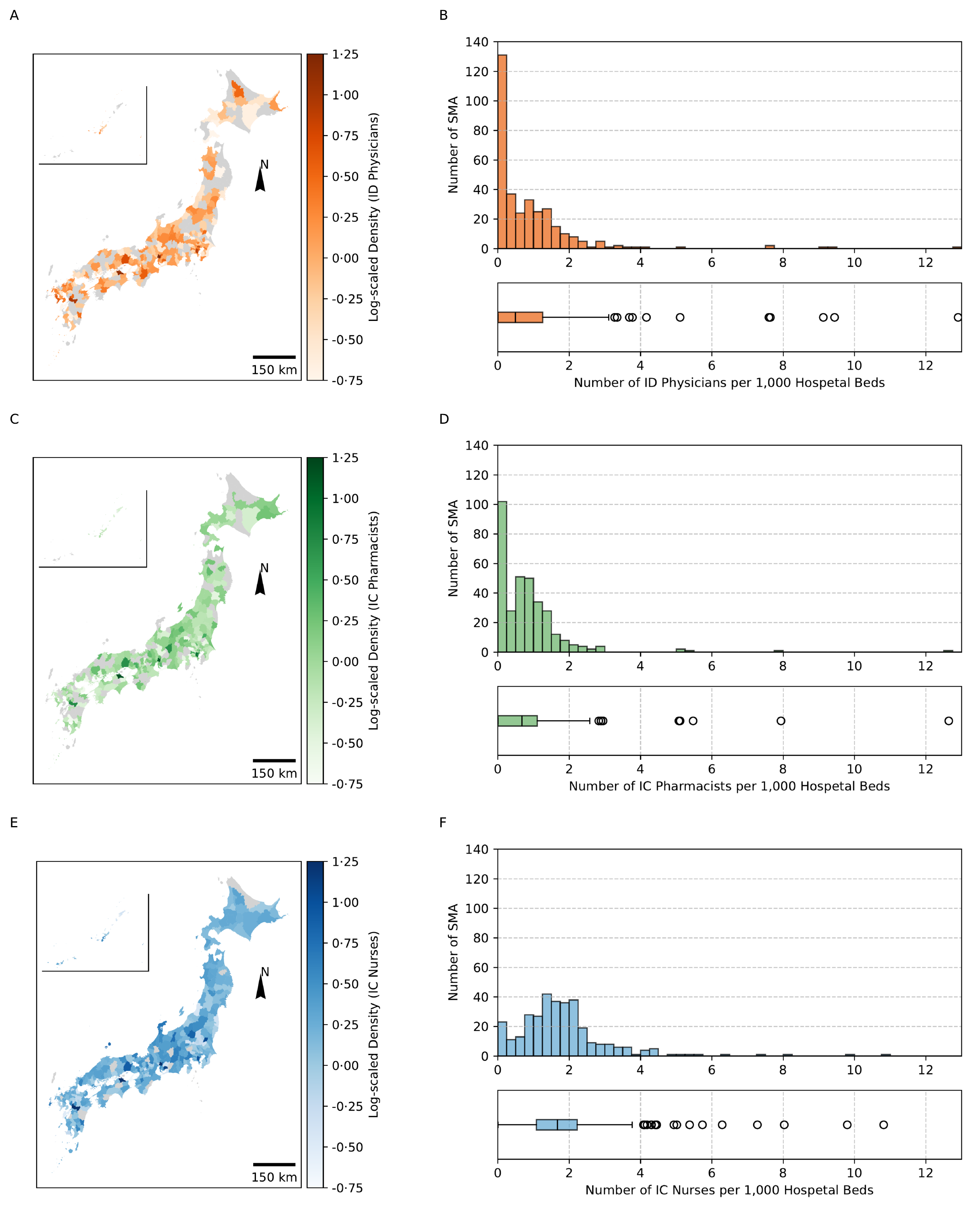

Supplement: Uesugi et al. supplementary material 2 — Uesugi et al. supplementary material [file S2732494X25102878sup002.tiff]

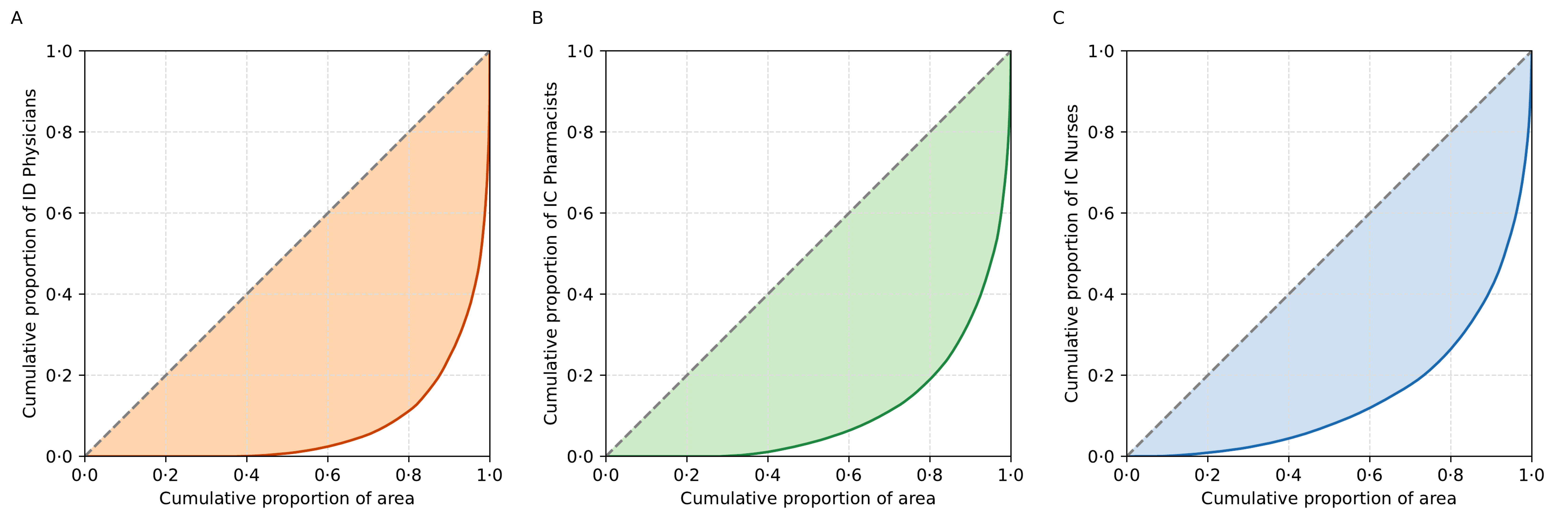

Supplement: Uesugi et al. supplementary material 3 — Uesugi et al. supplementary material [file S2732494X25102878sup003.tiff]

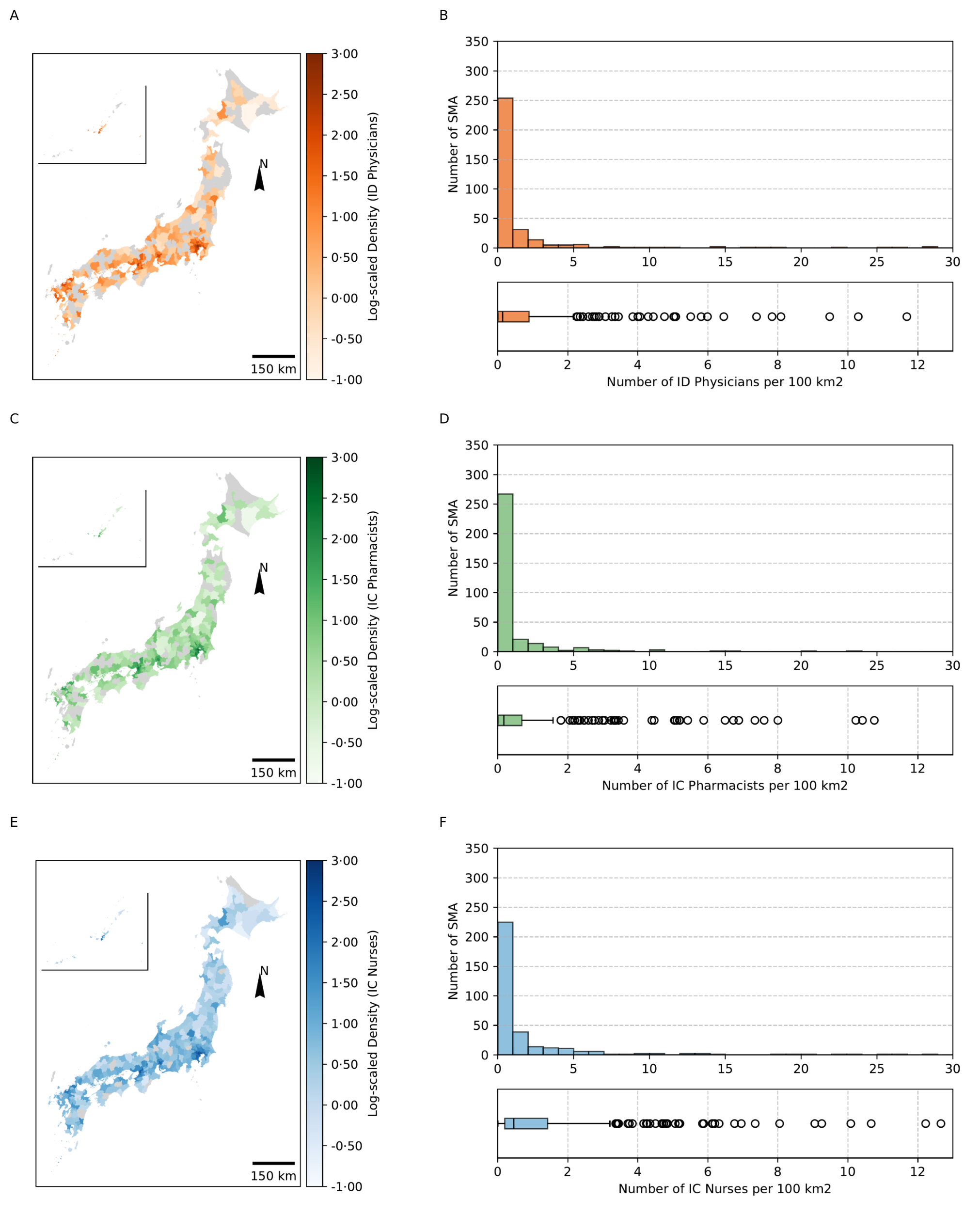

Supplement: Uesugi et al. supplementary material 4 — Uesugi et al. supplementary material [file S2732494X25102878sup004.tiff]
